# LncRNAs Target Ferroptosis-Related Genes and Impair Activation of CD4^+^ T Cell in Gastric Cancer

**DOI:** 10.3389/fcell.2021.797339

**Published:** 2021-12-13

**Authors:** Fuwen Yao, Yongqiang Zhan, Zuhui Pu, Ying Lu, Jiao Chen, Jing Deng, Zijing Wu, Binhua Chen, Jinjun Chen, Kuifeng Tian, Yong Ni, Lisha Mou

**Affiliations:** ^1^ Department of Hepatopancreatobiliary Surgery, Shenzhen Institute of Translational Medicine, Health Science Center, The First Affiliated Hospital of Shenzhen University, Shenzhen Second People’s Hospital, Shenzhen, China; ^2^ Shenzhen Xenotransplantation Medical Engineering Research and Development Center, Shenzhen Institute of Translational Medicine, Health Science Center, The First Affiliated Hospital of Shenzhen University, Shenzhen Second People’s Hospital, Shenzhen, China; ^3^ Imaging Department, Shenzhen Institute of Translational Medicine , Health Science Center, Shenzhen Second People’s Hospital, The First Affiliated Hospital of Shenzhen University, Shenzhen, China

**Keywords:** ferroptosis, CD4^+^ T cell activation, long non-coding RNAs, tumor microenvironment, gastric cancer, TCGA, immune infiltration, lncRNA

## Abstract

Gastric cancer (GC) is a malignant disease of the digestive tract and a life-threatening disease worldwide. Ferroptosis, an iron-dependent cell death caused by lipid peroxidation, is reported to be highly correlated with gastric tumorigenesis and immune cell activity. However, the underlying relationship between ferroptosis and the tumor microenvironment in GC and potential intervention strategies have not been unveiled. In this study, we profiled the transcriptome and prognosis data of ferroptosis-related genes (FRGs) in GC samples of the TCGA-STAD dataset. The infiltrating immune cells in GC were estimated using the CIBERSORT and XCELL algorithms. We found that the high expression of the hub FRGs (*MYB*, *PSAT1*, *TP53*, and *LONP1*) was positively correlated with poor overall survival in GC patients. The results were validated in an external GC cohort (GSE62254). Further immune cell infiltration analysis revealed that CD4^+^ T cells were the major infiltrated cells in the tumor microenvironment of GC. Moreover, the hub FRGs were significantly positively correlated with activated CD4^+^ T cell infiltration, especially Th cells. The gene features in the high-FRG score group were enriched in cell division, DNA repair, protein folding, T cell receptor, Wnt and NIK/NF-kappaB signaling pathways, indicating that the hub FRGs may mediate CD4^+^ T cell activation by these pathways. In addition, an upstream transcriptional regulation network of the hub FRGs by lncRNAs was also developed. Three lncRNAs (*A2M-AS1*, *C2orf27A*, and *ZNF667-AS1*) were identified to be related to the expression of the hub FRGs. Collectively, these results showed that lncRNA *A2M-AS1*, *C2orf27A*, and *ZNF667-AS1* may target the hub FRGs and impair CD4^+^ T cell activation, which finally leads to poor prognosis of GC. Effective interventions for the above lncRNAs and the hub FRGs can help promote CD4^+^ T cell activation in GC patients and improve the efficacy of immunotherapy. These findings provide a novel idea of GC immunotherapy and hold promise for future clinical application.

## 1 Introduction

Gastric cancer, as the third leading cause of cancer-related deaths, is responsible for a high burden of disease globally (Sung et al., 2021). In GC, ferroptosis is reported to be related to tumor cell proliferation and cancer therapeutic efficacy (Zhang et al., 2020; Ma et al., 2021). Ferroptosis, a type of programmed cell death, is induced by iron-dependent lipid peroxidation (Dixon et al., 2012). Moreover, increased ferroptosis is reported to contribute to the antitumor efficacy of immunotherapy (Wang et al., 2019a). Thus, in-depth research on ferroptosis in GC will help to clarify the understanding of tumorigenesis and develop effective approaches for GC treatment.

Recent studies have shown the relationship between ferroptosis and the immune microenvironment in the tumorigenesis and prognosis of GC (Zhang et al., 2020). The cells and molecules of the tumor microenvironment play a major role in tumor initiation and progression as well as responses to therapy (Voron et al., 2014; Subhash et al., 2015; Russick et al., 2020; Bejarano et al., 2021). The tumor microenvironment is composed of multiple components, such as macrophages, T cells, B cells, NK cells, tumor parenchymal cells, lymphocytes, fibroblasts, mesenchymal cells, and angiogenic factors (Subhash et al., 2015). Recently, it has been reported that CD4^+^ T cells might be a potential immunotherapeutic target for GC (Wei et al., 2018; Gu et al., 2020). Accordingly, CD4^+^ T cells are now commonly divided into two distinct lineages: Treg cells and conventional T helper cells (Corthay, 2009). Conventional Th cells control adaptive immunity by activating other effector cells, such as CD8^+^ cytotoxic T cells, B cells, and macrophages (Feau et al., 2012). Treg cells are defined as T cells in charge of suppression of potentially deleterious activities of Th cells, regulation of the effector class of the immune response, and suppression of T-cell activation (Corthay, 2009; Arumugam and Sugin Lal Jabaris, 2021). The study of CD4^+^ T cells has tremendous potential to contribute to cancer immunotherapy (Yuan et al., 2017). It has been previously reported that ferroptosis is involved in regulating CD4^+^ T cell homeostasis, which enhances the function of follicular helper T cells during infection and following vaccination (Yao et al., 2021). However, the function of ferroptosis in the regulation of the GC microenvironment and the upstream regulators of ferroptosis-related genes (FRGs) are worth further exploration.

In this study, we identified four important FRGs (the hub FRGs: *PSAT1*, *MYB*, *TP53*, and *LONP1*) that were related to gastric carcinogenesis and prognosis. Based on immune cell infiltration analysis of different immune cell types, we found that the expression of the hub FRGs was negatively correlated with resting CD4^+^ T cell infiltration but positively correlated with activated CD4^+^ T cell infiltration in the GC samples of the TCGA-STAD dataset. We indicated that the hub FRGs might participate in CD4^+^ T cell activation. The T cell receptor signaling pathway, NIK/NF-kappaB signaling pathway and Wnt signaling pathway were shown to be significantly related to the hub FRGs. To assess the potential upstream regulatory mechanism of the hub FRGs, we identified three lncRNAs (*A2M-AS1*, *C2orf27A*, and *ZNF667-AS1*) that might inhibit the expression of the hub FRGs. Collectively, these results revealed that these three lncRNAs are upstream regulators of the hub FRGs and affect the GC microenvironment.

## 2 Materials and Methods

### 2.1 Data Preparing and Processing

Ferroptosis-related genes (FRGs): We first obtained 289 FRGs from previous studies (PMID: 33425905, 31634899, 31634900, 31105042, 28985560, and 32760210) (Stockwell et al., 2017; Bersuker et al., 2019; Doll et al., 2019; Hassannia et al., 2019; Liu et al., 2020a; Liang et al., 2020).

TCGA-STAD dataset: The gene expression data of 375 GC patients and 32 adjacent cancer samples and clinical data of matched patients were obtained from The Cancer Genome Atlas (TCGA) data portal (https://TCGAData.nci.nih.gov/TCGA/). For differential expressed gene (DEG) analysis of mRNA, we used the limma package in R 4.0.5, and genes with a *p*-value <0.05 and fold change >1.5 were selected to be differentially expressed. For Kaplan-Meier (K-M) analysis and Cox regression analysis, a risk score was calculated for each patient, and a median value was identified for all patients. GC patients were then divided into a low group (score below the median) and a high group (score above the median). The high and low groups were stratified and visualized using K-M survival curves and analyzed for statistical significance using the log-rank test. Cox regression analysis and K-M curves with the log-rank test were conducted by the glmnet and survival packages in R 4.0.5.

GSE62254 dataset (validation dataset): K-M analysis of the GEO dataset (GSE62254) was performed by the Kaplan-Meier Plotter database (https://kmplot.com/analysis/) (Cristescu et al., 2015). We used the Auto select best cutoff function to split patients.

LncRNAs: LncRNAs were annotated by GENCODE (version 25). We used the limma package in R 4.0.5 to perform DEG analysis. LncRNAs with a *p*-value <0.05 and fold change >1.5 were selected to be significantly differentially expressed. K-M analysis and Cox regression analysis were used to calculate median survival.

The heatmap plots were displayed by the scaled FPKM of each gene across the whole GC samples.

### 2.2 Correlation Analysis of Gene Expression

We applied Pearson’s correlation test to assess the correlations between the hub FRGs and other genes. Pearson’s correlation test was performed in R 4.0.5. Gene pairs with Pearson’s correlation coefficient >0.1 and *p*-value <0.01 will be considered coexpressed genes.

### 2.3 Immune Infiltration Analysis

The CIBERSORT and XCELL algorithms, which enable estimation of cell type abundances from bulk tissue transcriptomes, were analyzed to assess the percentage of immune cell types. We applied CIBERSORT and XCELL methods in TIMER (http://timer.comp-genomics.org/) to infer the infiltration fraction of different types of immune cells among GC samples in the TCGA-STAD dataset based on the FPKM of genes in GC samples.

For the correlation between the hub FRGs and immune cell markers, we used the correlation function in TIMER (https://cistrome.shinyapps.io/timer/). The TCGA-STAD dataset was selected for the correlation analysis.

### 2.4 Gene Ontology Enrichment Analysis

GO terms of selected genes were enriched using DAVID (the database for annotation, visualization, and integrated discovery, https://david.ncifcrf.gov/), which is an online tool for functional annotation and enrichment analysis to reveal biological features related to large gene lists. The visualization of representative biological process GO terms was performed by the ggplot2 package in R4.0.5.

### 2.5 Long Non-Coding RNAs-Hub Ferroptosis-Related Genes Network Analysis

LncRNAs were annotated by GENCODE (version 25) (https://www.gencodegenes.org/). The correlation between lncRNAs and the hub FRGs was calculated by Pearson’s correlation analysis. The connectivity of a given gene was measured by the sum of absolute values of Pearson’s correlation coefficient between lncRNAs and the hub FRGs. The red color represents more connectivity between two genes.

### 2.6 Statistical Analyses

The statistical analyses, including t-tests and log-rank tests, were performed using R 4.0.5. The limma package in R 4.0.5 was used to detect DEGs with the cutoff of *p*-value <0.05 and fold change >1.5. Data visualization was performed by the ggplot2 package in R 4.0.5. *p* < 0.05 was considered to be statistically significant.

## 3 Results

### 3.1 Ferroptosis-Related Genes Play Crucial Roles in Gastric Carcinogenesis and Progression

**FIGURE 1:**
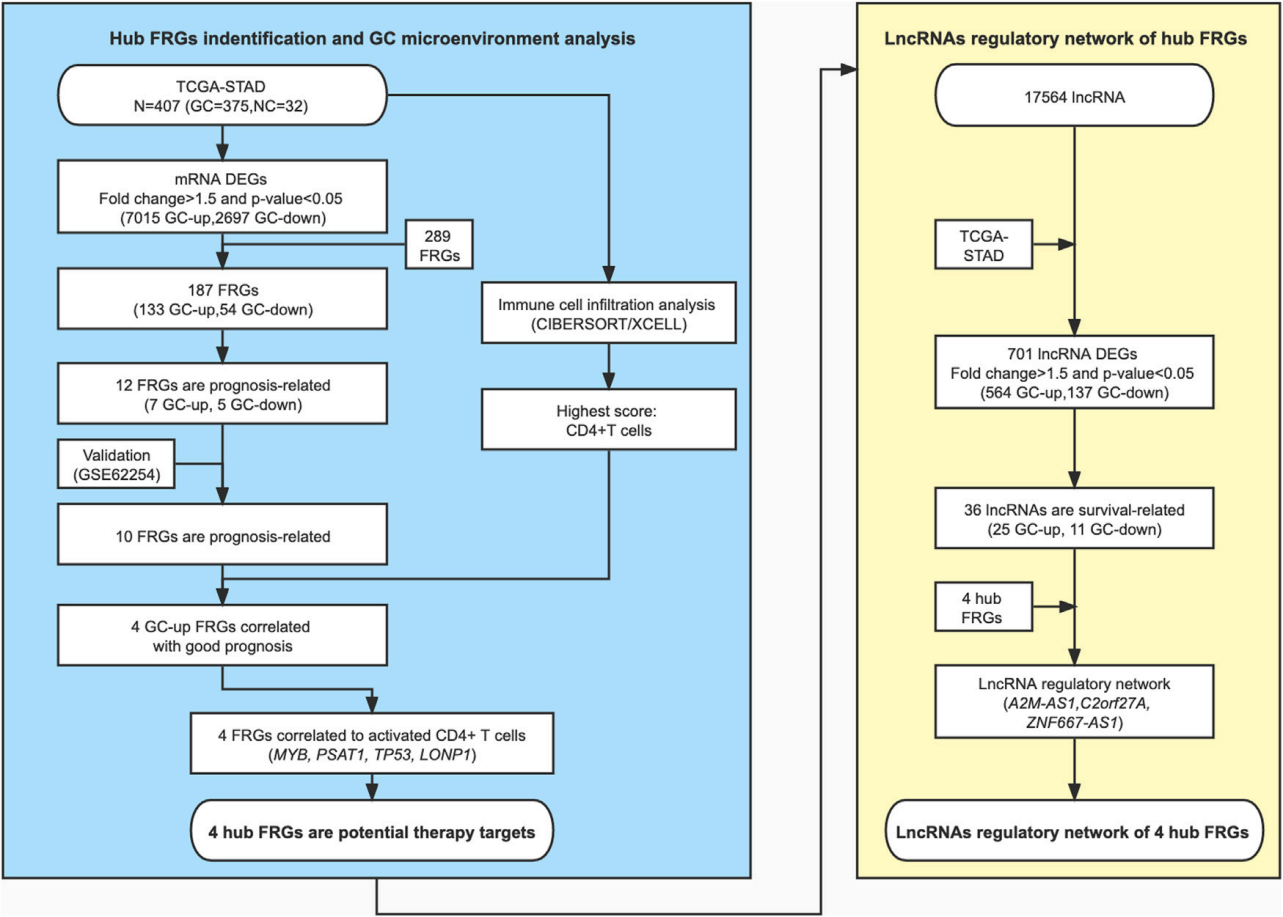
A schematic diagram of the study design and patient cohorts for the identification of hub ferroptosis-related genes.

**FIGURE 2:**
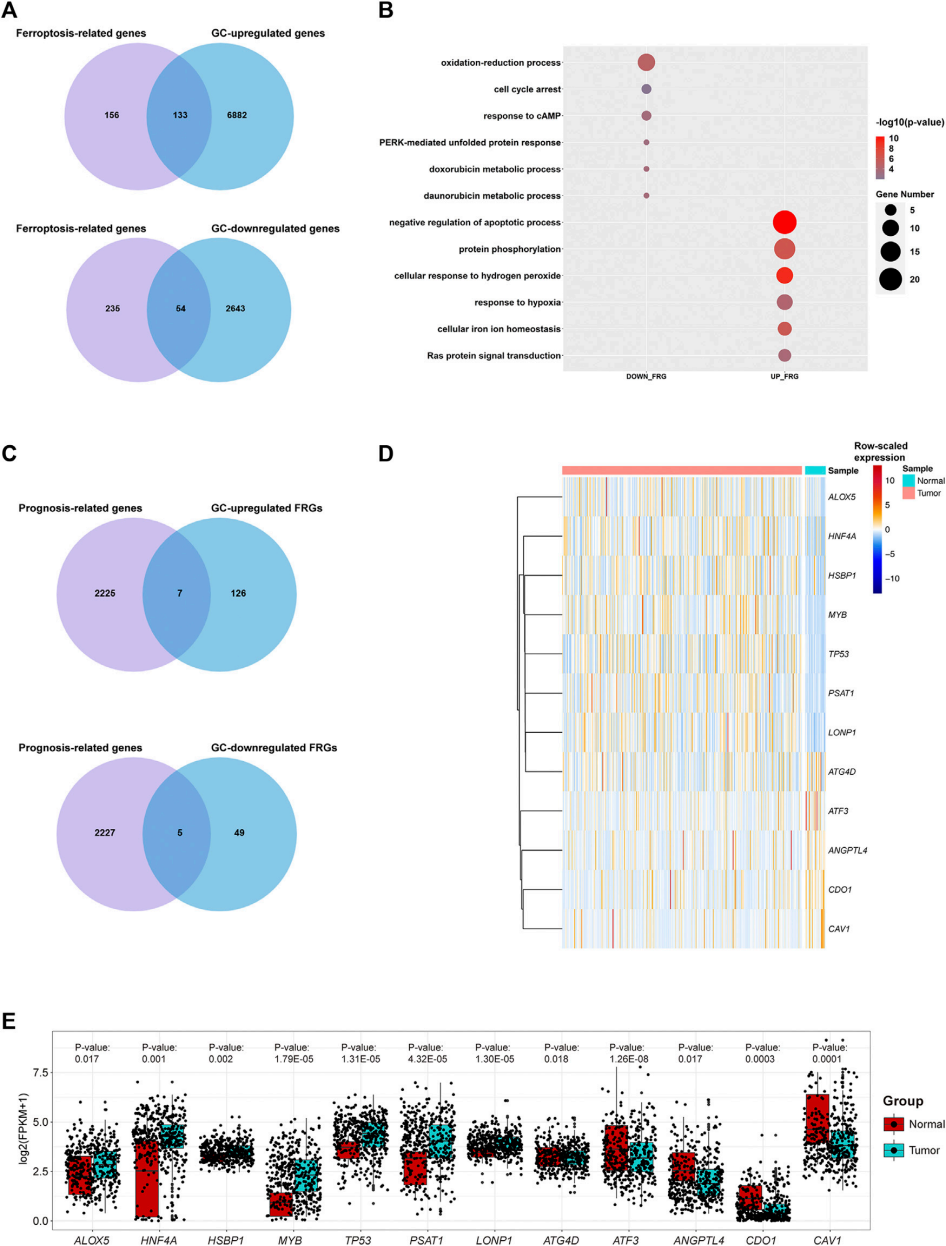
Transcriptome profiling of gastric cancer samples in the TCGA dataset. **(A)** Venn diagram representation of ferroptosis-related genes (FRGs) and differentially expressed genes (DEGs) between GC samples and adjacent cancer samples in the TCGA dataset. **(B)** Representative GO terms of GC-differentially expressed FRGs in the TCGA dataset. **(C)** Venn diagram representation of prognosis-related genes and GC-differentially expressed FRGs in the TCGA dataset. **(D)** Heatmap plot for raw-scaled expression of important FRGs in GC samples and adjacent cancer samples in the TCGA dataset. **(E)** Boxplot for the expression (log(FPKM+1)) of the 12 differentially expressed FRGs.

We show the workflow chart of this study in Figure 1. To identify the key genes involved in the process of gastric carcinogenesis as well as prognosis, we conducted an in-depth analysis of public RNA-Seq data from the TCGA-STAD dataset of 375 gastric cancer (GC) and 32 adjacent normal tissues. Based on the expression profile in the GC samples of the TCGA-STAD dataset, 9,712 differentially expressed genes (DEGs) were analyzed in GC tissues compared with adjacent normal tissues (fold change >1.5, *p*-value <0.05) (Figure 2A; Supplementary Table S1). Among these DEGs, 7015 were upregulated in GC and 2,697 were downregulated in GC (Figure 2A). To assess the expression of FRGs in gastric carcinoma and to determine their roles in gastric carcinogenesis, we obtained 289 FRGs from previous studies (Stockwell et al., 2017; Bersuker et al., 2019; Doll et al., 2019; Hassannia et al., 2019; Liu et al., 2020a; Liang et al., 2020) (Supplementary Table S2). Nearly 65% (187 of 289) of FRGs were differentially expressed between GC tissues and adjacent mucosa in the TCGA-STAD dataset (Figure 2A; Supplementary Table S3). Among them, 133 FRGs were GC-upregulated, and 54 FRGs were GC-downregulated (Figure 2A; Supplementary Table S3). GO enrichment analysis of the above FRGs revealed multiple significantly enriched signaling pathways associated with GC, as well as crucial pathways related to ferroptosis. GC-upregulated FRGs were significantly enriched in the cellular response to hydrogen peroxide and cellular iron ion homeostasis pathways, whereas GC-downregulated FRGs were enriched in the oxidation-reduction process, response to cAMP, and cell cycle arrest GO terms, indicating the crucial roles of ferroptosis in GC (Figure 2B; Supplementary Table S4).

**FIGURE 3:**
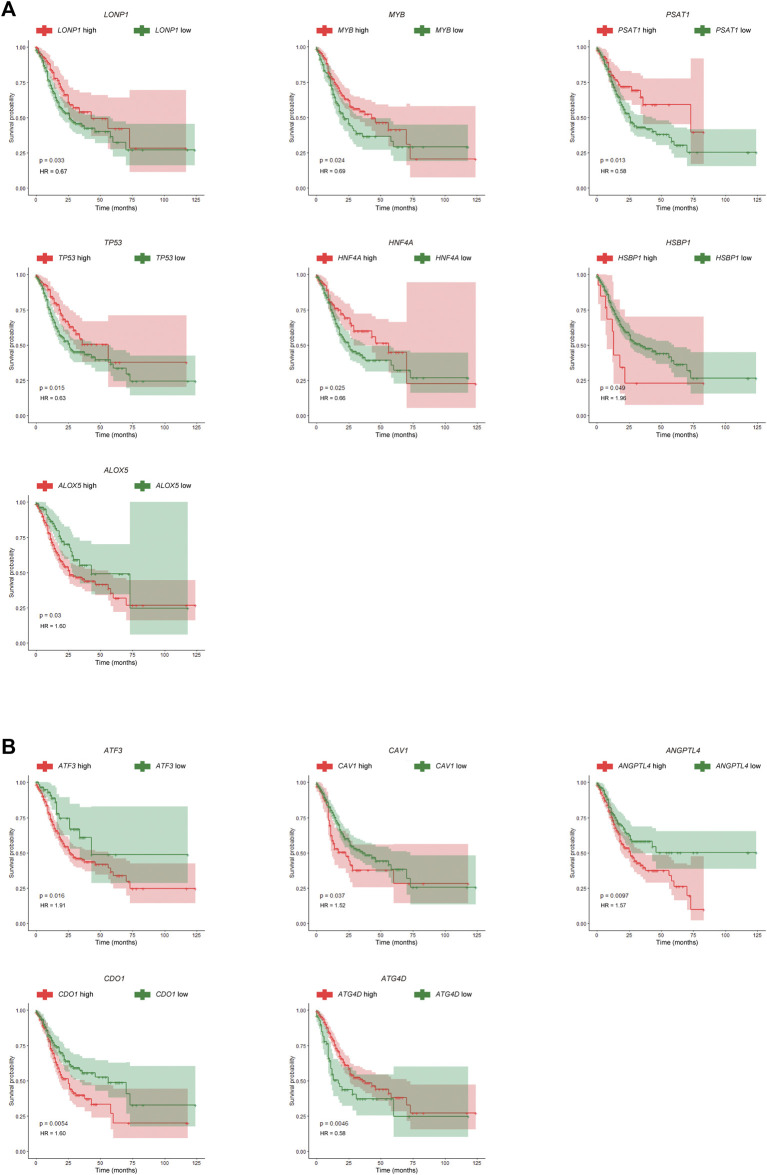
Kaplan-Meier (K-M) survival curves for prognosis-related FRGs in GC. **(A)** K-M survival curves and *p*-values for the seven GC-upregulated and prognosis-related FRGs. **(B)** K-M survival curves and *p*-values for the five GC-downregulated and prognosis-related FRGs.

Furthermore, seven differentially expressed FRGs were significantly associated with good prognosis, and five differentially expressed FRGs were significantly associated with poor prognosis (Figure 2C). The expression levels of these 12 genes in the TCGA-STAD dataset were shown in Figures 2D,E. The TCGA-STAD cohort was stratified into high and low groups according to the median expression level of each gene, and all 12 genes were significantly correlated with prognosis (*p* < 0.05, Figures 3A,B). Among them, seven GC-upregulated FRGs (*LONP1*, *MYB*, *PSAT1*, *TP53*, *HNF4A*, *HSBP1*, and *ALOX5*) and five GC-downregulated FRGs (*ATF3*, *CAV1*, *ANGPTL4*, *CDO1*, and *ATG4D*) were prognosis-related genes (Figures 3A,B). To validate these results of the GC prognosis-related FRGs, we analyzed another transcriptome dataset (GSE62254) of GC with corresponding survival data (Cristescu et al., 2015). The results showed that 10 (*LONP1*, *MYB*, *PSAT1*, *TP53*, *HNF4A*, *ALOX5*, *ATF3*, *CAV1*, *ANGPTL4*, and *CDO1*) of these 12 FRGs were also significantly correlated with prognosis in the GSE62254 dataset (Supplementary Figure S1), indicating that these 10 FRGs may influence ferroptosis and GC progression.

### 3.2 Four Hub Ferroptosis-Related Genes May be Related to CD4^+^ T Cell Activation

**FIGURE 4:**
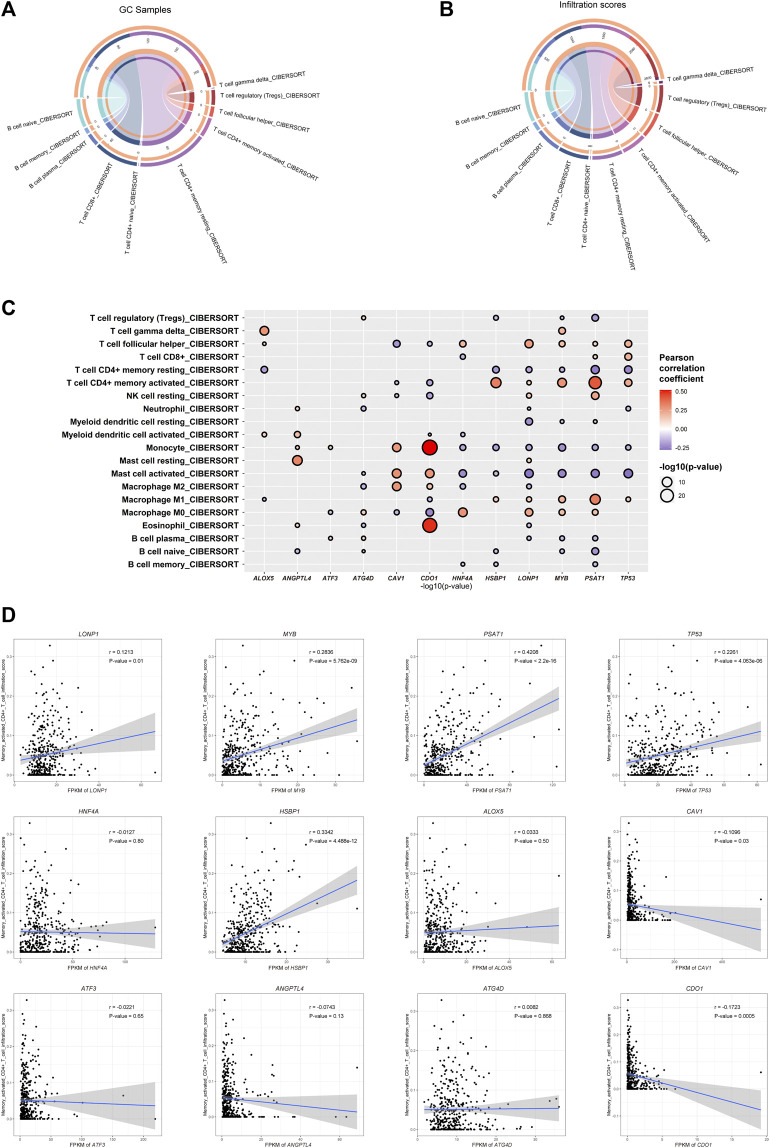
Ferroptosis-related genes are related to immune cell infiltration. **(A)** Counts of each infiltrated immune cell type in GC. **(B)** Total infiltrated scores of each infiltrated immune cell type in GC. **(C)** Correlation between the infiltration scores of each immune cell type and the expression of GC-differentially expressed and prognosis-related FRGs. **(D)** Dot plot showing the positive correlation between the hub FRGs (*MYB*, *LONP1*, *TP53*, and *PSAT1*) and infiltration scores of CD4^+^ T cells.

To investigate the relationship between the important FRGs and the tumor microenvironment of GC, we calculated the different immune cell infiltration scores of patients in the TCGA-STAD dataset based on CIBERSORT. We found that the infiltrated immune cells in the TCGA-STAD dataset were mainly CD4^+^ memory T cells, CD8^+^ T cells, naïve B cells, Tregs, and so on. Among them, CD4^+^ T cells, including Tregs, follicular helper T cells, activated memory CD4^+^ T cells, resting memory CD4^+^ T cells, and naïve CD4^+^ T cells, had the highest immune cell infiltration score and the largest number of infiltrated patients (Figures 4A,B; Supplementary Table S5). The main type of infiltrated immune cells in GC patients might be CD4^+^ T cells. Among them, resting or activated memory CD4^+^ T cells accounted for a large proportion. Subsequently, we explored the Pearson’s correlation coefficient of the expression of prognosis-related FRGs and different immune cell infiltration scores. Interestingly, we found that most GC prognosis-related FRGs were significantly correlated with one or more immune cell infiltration scores (Figure 4C). More interestingly, four of the GC-upregulated FRGs (*LONP1*, *MYB*, *PSAT1*, and *TP53*) showed a significant positive correlation with activated CD4^+^ T cell infiltration (Pearson’s correlation coefficient >0.1 and *p*-value ≤ 0.01, Figures 4C,D; Supplementary Table S6). This indicates that during gastric carcinogenesis, high expression of the hub FRGs (*PSAT1*, *LONP1*, *MYB*, *TP53*) may promote an increase in activated CD4^+^ T cells. Since CD4^+^ T cells help for recruitment, proliferation, and effector function of CD8^+^ T cells (Bos and Sherman, 2010; Ahrends et al., 2019), we speculate that activated CD4^+^ T cells could promote the killing efficiency of tumor cells by CD8^+^ T cells in GC. Our study further showed that the expression of the hub FRGs was negatively correlated with the infiltration level of resting CD4^+^ T cells (Figure 4C), which suggests that the hub FRGs may promote the conversion of CD4^+^ T cells from resting to activated, thereby affecting the prognosis of GC patients.

We found that the gene expression of the hub FRGs was significantly related to the marker genes of some CD4^+^ T cells (Supplementary Figure S2A). Due to the diversity of CD4^+^ T cell types, we further selected markers of different types of CD4^+^ T cells to explore the correlation between the hub FRGs and the expression of markers of different types of CD4^+^ T cells. We found that the expression of *TP53* was significantly positively correlated with *CCR8*, which is a member of the beta chemokine receptor family and serves as one of the Th2 cell markers. *LONP1* was positively correlated with *IL21R*, which is an important molecule involved in many cytokine-induced immune and inflammatory responses and acts as a Th17 cell marker. *TP3* is significantly positively correlated with *KLRD1*, *CXCR3*, *CXCR6*, *CCR5*, and *IL12RB1*, which serve as Th1 cell markers. Other important co-expression gene pairs also showed a strong relationship among the hub FRGs and multiple markers of different types of CD4^+^ T cells. In addition, we found that *CD25*, the main marker of Tregs, did not show much correlation with *PSAT1*, *LONP1*, and *MYB*, indicating that Th cells, not Tregs, were related to the expression of the hub FRGs (Supplementary Figure S2B). To further prove this, we calculated the infiltration scores of cell types of GC using the XCELL algorithm based on the expression data of GC samples in the TCGA-STAD dataset, which allowed us to identify more infiltrated cell types of GC. We also found that the infiltration scores of Th1 (25.1) and Th2 (31.3) cells were much higher than that of Tregs (4.1), indicating that the most dominant types of infiltrated CD4^+^ T cells in GC are Th1 and Th2 cells, instead of Tregs (Supplementary Figure S2C). In addition, a number of cancer-associated fibroblasts (CAFs) and endothelial cells were also observed, which suggested diverse cell types in the tumor microenvironment of GC. Therefore, we demonstrated that the hub FRGs might promote the activation of different types of CD4^+^ T cells, especially Th cells, thereby promoting a good prognosis for GC patients.

### 3.3 Subgroup Analysis of the Hub Ferroptosis-Related Genes, Tumor Microenvironment, and Clinical Characteristics

**FIGURE 5:**
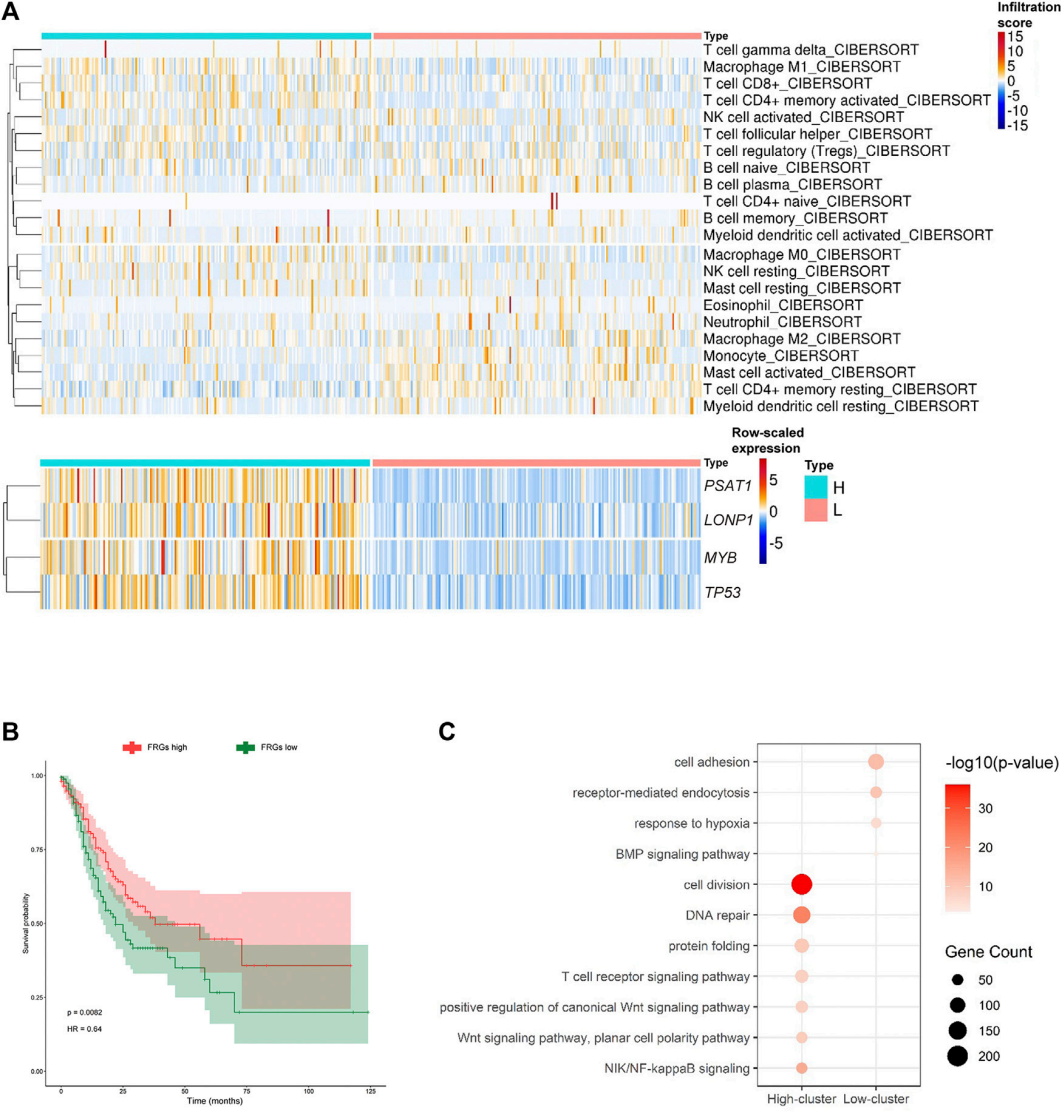
*MYB*, *LONP1*, *TP53*, and *PSAT1* are related to CD4^+^ T cell activation. **(A)** Heatmap showing the infiltration scores of each immune cell type in the two subgroups of GC divided by the average expression of the hub FRGs (high-FRG score and low-FRG score group). **(B)** K-M survival curves and *p*-values for the two subgroups divided by the average expression of the hub FRGs. **(C)** Representative GO terms enriched by DEGs between the two subgroups divided by the average expression of the hub FRGs.

We initially realized that the hub FRGs may promote the activation of CD4^+^ T cells, and the activated CD4^+^ T cells may correlate with good prognosis of GC patients. To further explore the effects of the hub FRGs on the classification, prognosis, and treatment of GC samples, we classified TCGA-STAD GC patients into high-FRG score and low-FRG score groups according to the average expression levels of the hub FRG genes (Figure 5A; Supplementary Table S7). Additionally, to evaluate the relationship between FRGs and the tumor microenvironment, we used the CIBERSORT algorithm to examine the proportions of immune cells. The infiltration score for each patient was calculated, and the results showed that the resting CD4^+^ T cell infiltration scores in the high-FRG score group were significantly lower than those in the low-FRG score group. The activated CD4^+^ T cell infiltration scores in the high-FRG group score were significantly higher than those in the low-FRG score group (Figure 5A). Furthermore, the Kaplan-Meier curve indicated that the high-FRG group score had a significantly longer overall survival time than the low-FRG score group (Figure 5B).

### 3.4 Identification of Pathways Related to CD4^+^ T Cell Activation by the Hub Ferroptosis-Related Genes

To identify the pathways related to CD4^+^ T cell activation by the hub FRGs, we analyzed the enriched pathways of GO terms in the high-FRG score group and low-FRG score group. The highly expressed genes in the high-FRG score group were significantly enriched in cell division, DNA repair, protein folding, T cell receptor, Wnt and NIK/NF-kappaB signaling pathways. In contrast, the low-FRG score group was significantly enriched in cell adhesion, receptor-mediated endocytosis, response to hypoxia, and the BMP signaling pathway (Figure 5C; Supplementary Table S8). As a result, it was possible that the hub FRGs mediated CD4^+^ T cell activation by cell division, DNA repair, protein folding, T cell receptor, Wnt and NIK/NF-kappaB signaling pathways.

### 3.5 Long Non-Coding RNAs can Regulate the Hub Ferroptosis-Related Genes and Act as Potential Intervention Targets

**FIGURE 6:**
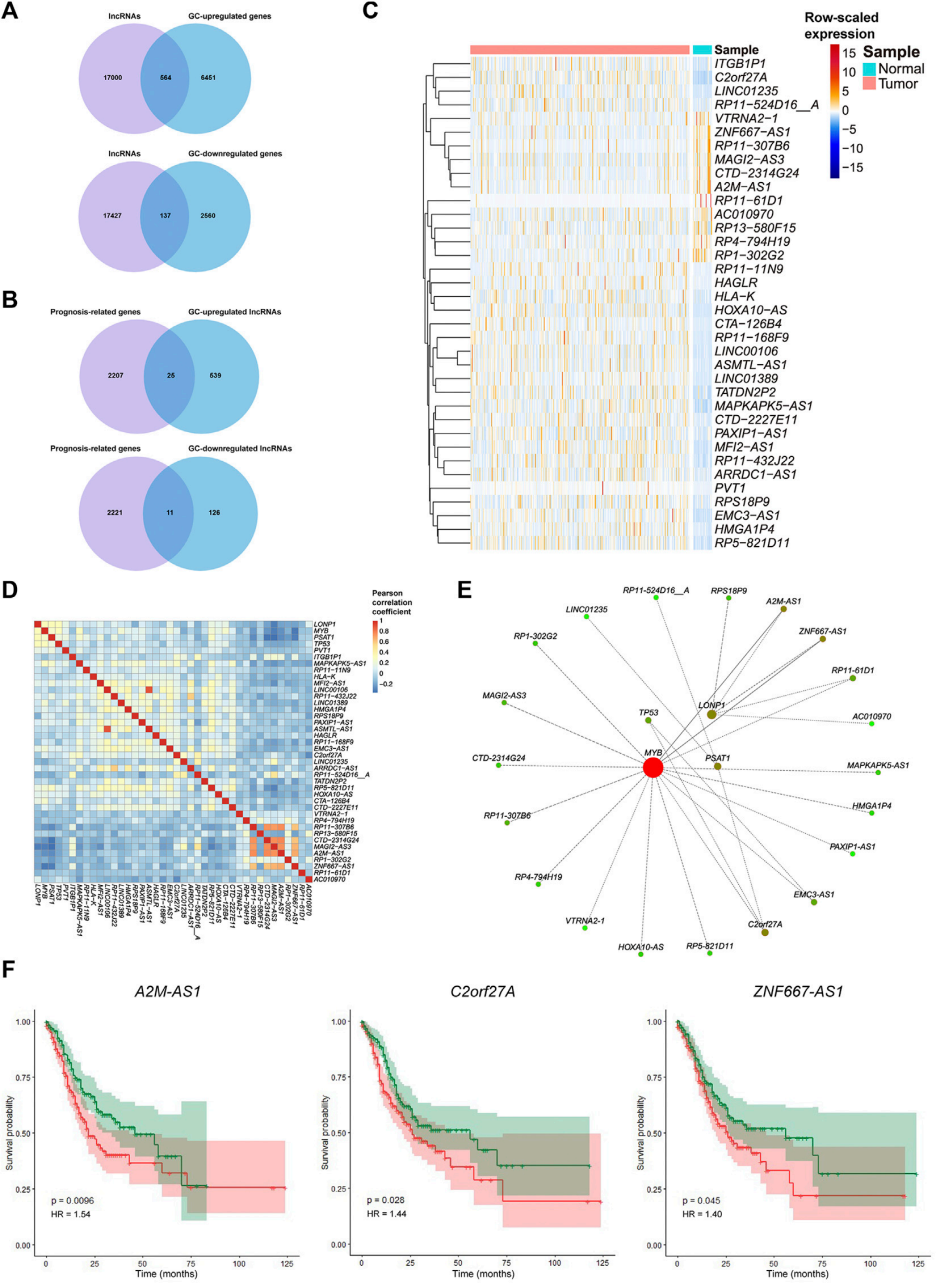
Relationships of the expression of the hub FRGs and their correlated lncRNAs involved in tumorigenesis and prognosis of GC. **(A)** Venn diagram representation of lncRNAs obtained from GENCODE (version 25) and DEGs between GC samples and adjacent cancer samples in the TCGA dataset. **(B)** Venn diagram representation of prognosis-related genes and GC-differentially expressed lncRNAs in the TCGA dataset. **(C)** Heatmap plot for the expression of important lncRNAs in GC samples and adjacent cancer samples in the TCGA dataset. **(D)** Correlation heatmap for Pearson’s coefficient of the expression of important lncRNAs and hub FRGs. **(E)** Interaction network of important lncRNAs and hub FRGs based on their connectivity. Red represents more connectivity between two genes. **(F)** K-M survival curves and *p*-values for the three important lncRNAs (*A2M-AS1*, *C2orf27A*, and *ZNF667-AS1*). Patients with high or low expression of one of the three lncRNAs are labeled with a red line or green line, respectively.

We identified the hub FRGs that might contribute to the good prognosis of GC by promoting the activation of CD4^+^ T cells. However, their potential upstream regulation mechanism is still unknown. As the “dark matter of the genome,” long noncoding RNAs (lncRNAs) have been reported to regulate gene expression through recruiting regulatory complexes, interacting with RNA binding proteins, interfering with transcription, and other mechanisms (Chang and Han, 2016; Shankaraiah et al., 2018; Gao et al., 2021). To investigate the possible regulatory mechanisms of the hub FRGs and lncRNAs, we further analyzed the lncRNAs related to the hub FRGs based on Pearson’s correlation coefficient of gene expression. We first screened the expression of 17,564 lncRNAs (from GENCODE, version 25) in the TCGA-STAD dataset. The results showed that 701 lncRNAs were differentially expressed (564 were significantly upregulated and 137 were downregulated in the GC group) (Figure 6A). A total of 36 of them were significantly related to the prognosis of GC patients (25 GC upregulated genes and 11 GC downregulated genes) (Figures 6B,C; Supplementary Table S9). Subsequently, we constructed a lncRNA-FRG interaction network based on the connectivity of genes in the interaction network (Figures 6D,E). Among them, three lncRNAs with the strongest regulatory relationship with the hub FRGs were *A2M-AS1*, *C2orf27A*, and *ZNF667-AS1* (Figure 6E). Since they were also negatively correlated with activated CD4^+^ T cell infiltration (Supplementary Figure S3), we demonstrated that the three lncRNAs might potentially negatively regulate the hub FRGs, which in turn inhibit the activation of CD4^+^ T cells. High expression of these three lncRNAs was associated with poor prognosis in GC patients (Figure 6F). Therefore, lncRNAs *A2M-AS1*, *C2orf27A*, and *ZNF667-AS1* may serve as potential lncRNA targets for regulating ferroptosis, CD4^+^ T cell activation, and the prognosis of GC. Effective interventions can help promote CD4^+^ T cell activation in GC patients and improve the efficacy of immunotherapy.

## 4 Discussion

In this study, we provide a comprehensive transcriptome-wide analysis of ferroptosis-related genes (FRGs) in gastric cancer (GC). Twelve GC-differentially expressed and prognosis-related FRGs were identified as important genes in the TCGA-STAD dataset. Ten of these twelve GC-differentially expressed and prognosis-related FRGs were further validated in the GSE62254 dataset. Interestingly, the high expression of the good-prognosis-related hub FRGs (*PSAT1*, *MYB*, *TP53*, and *LONP1*) was positively correlated with activated CD4^+^ T cell infiltration, while negatively correlated with resting CD4^+^ T cell infiltration in the TCGA-STAD dataset. Thus, we suggest that the hub FRGs have the potential to be involved in CD4^+^ T cell activation, especially Th cells. The activated Th cells may promote antitumor immunity in GC (Dadaglio et al., 2020). *PSAT1* (phospholipid sterol acyltransferase 1) has been reported to be critically important for the transition from p-Pyr to p-Ser (Mullarky et al., 2016) and further affects ferroptosis (Zhang et al., 2019). *MYB* (C-Myb) can interact directly with *CDO1* and play a significant role in ferroptosis (Hao et al., 2017). *TP53* serves as a vital factor in cellular ferroptosis in response to various damages (Wang et al., 2016; Wang et al., 2019b). *LONP1* has also been reported to be involved in ferroptosis by regulating *GPX4* (Wang et al., 2020a). It has been previously reported that ferroptosis is involved in regulating CD4^+^ T cell homeostasis (Yao et al., 2021). Our results further revealed that the hub FRGs mainly regulate the T cell receptor signaling pathway, NIK/NF-kappaB signaling pathway, and Wnt signaling pathway. To our knowledge, NIK/NF-kappaB regulates the balance of different types of CD4^+^ T cells (Rowe et al., 2013). NIK/NF-kappaB activation is also involved in T helper (Th) cell differentiation, including Th17 cell differentiation (Park et al., 2014). The Wnt signaling pathway is essential for activating CD4^+^ T cells and restoring immune homeostasis (Schenkel et al., 2010; Sun et al., 2017). Although these three significant pathways might be the mechanisms of the activation of CD4^+^ T cells induced by the hub FRGs in GC, further studies are needed to provide conclusive evidence for the specific mechanisms in the tumor microenvironment (TME).

The TME plays a crucial role in the progression and treatment outcomes of tumors. However, the establishment and function of the TME remain obscure due to its complex cellular composition (Yao et al., 2020). The TME consists of multiple cell types, including blood vessels, fibroblasts, immune cells, and other tissue-resident cells (Dai et al., 2017). In addition, the immune status of the TME is extremely complex (Paudel et al., 2019). It has been reported that the infiltration of cytotoxic T cells and memory T cells was associated with good prognosis in gastric patients (Chang et al., 2014). Although the current tumor immunotherapy targets mainly focus on CD8^+^ T cells, the role of conventional CD4^+^ T cells in tumor immunity has gradually attracted attention (Nishikawa and Koyama, 2021). CD4^+^ effector T cells can enhance immunity by regulating dendritic cells or stimulating other proinflammatory cells of the myeloid lineage (Behrens et al., 2004). Since we found that CD4^+^ T cells are the main type of infiltrated immune cells in GC, our study mainly investigates the regulatory relationships and potential mechanism linking FRGs and CD4^+^ T cell activation in GC. Our results showed that activation of some subtypes of CD4^+^ T cells, especially Th1 cells, can promote T cell responses and enhance the effect of immunotherapy.

We demonstrated that FRGs play crucial roles in CD4^+^ T cell activation. However, the lack of knowledge of CD4^+^ T cells in immunotherapy limits their application. In this study, we found that the activation of Th cells, instead of Tregs, is related to the hub FRGs. The activation of Th cells can provide cytokines to support important regulatory and effector functions of T cells (Subhash et al., 2015). Since *PSAT1*, *MTB*, *TP53*, and *LONP1* were all suggested to be related to CD4^+^ T cell activation, especially Th cells, we speculate that they have the potential for adjuvant immunotherapy based on CD4^+^ T cells and improve survival in patients with GC. This further supports the suggestion that these genes have the potential to serve as targets for adjuvant immunotherapy.

We constructed the upstream regulatory network of the hub FRGs by lncRNA. Three lncRNAs (*A2M-AS1*, *C2orf27A*, and *ZNF667-AS1*) were identified to be related to the hub FRGs. Among them, *A2M-AS1* was reported to be involved in the invasion, migration, and progression of breast cancer (Fang et al., 2020; Liu et al., 2020b). In addition, *A2M-AS1* was also reported to lessen cardiomyocyte injury caused by hypoxia/reoxygenation (H/R) by regulating *IL1R2* (Song et al., 2020). Since hypoxia and reoxygenation are also reported to be related to ferroptosis (Li et al., 2020; Eleftheriadis et al., 2021), our study provides a further potential mechanism of *A2M-AS1*, ferroptosis, and even H/R. *C2orf27A* is reported to be associated with sorafenib resistance, clinical cancer stages, and pathological tumor grades in liver cancer (Yuan et al., 2021). Our study confirms the role of *C2orf27A* in tumor progression and provides a new view of it in gastric carcinogenesis and prognosis, as well as its potential relationship with FRGs. *ZNF667-AS1* is also a tumor-related lncRNA. Previous studies have reported that *ZNF667-AS1* can suppress the progression of nasopharyngeal carcinoma colorectal cancer and cervical cancer (Li et al., 2019; Chen et al., 2020; Zhuang et al., 2021). However, there are also some reports that indicate that *ZNF667-AS1* can inhibit the inflammatory response and promote liver metastasis in acute myeloid leukemia (AML) (Wang et al., 2020b). We further identified that it can promote the progression of GC, probably by regulating FRGs and CD4^+^ T cell activation. These studies provide strong evidence of our lncRNA regulatory network analysis. Their biological roles in GC need further experimental validation. Thus, the regulation of these essential lncRNAs has the potential to act as a novel intervention strategy for antitumor immunotherapy mediated by enhanced CD4^+^ T cell activation, especially Th cells.

Taken together, our study indicates the relationship between ferroptosis and the TME in GC. We propose that ferroptosis is related to CD4^+^ T cell activation in GC. We identified three lncRNAs were predicted to regulate the hub FRGs. The hub FRGs (*PSAT1*, *MYB*, *TP53*, and *LONP1*) and three lncRNAs (*A2M-AS1*, *C2orf27A*, and *ZNF667-AS1*) have the potential to serve as novel targets for adjuvant immunotherapy. Further studies on ferroptosis-induced CD4^+^ T cell activation will provide a new perspective on the complexity of the TME and diverse strategies for cancer immunotherapy.

## Data Availability

The original contributions presented in the study are included in the article/Supplementary Material, further inquiries can be directed to the corresponding authors.

## Supplementary Material

Supplementary Figure 1 K-M survival curves and *p*-values for the 12 GC-differentially expressed and prognosis-related FRGs based on the GSE62254 dataset.

Supplementary Figure 2 Correlation between expression of important FRGs and markers of different types of CD4^+^ T cells. **(A)** Correlation between the expression of important FRGs and classical markers of CD4^+^ T cells. **(B)** Correlation between the expression of important FRGs and markers of Tfh cells, Th1 cells, Treg cells, Th2 cells, and Th17 cells. **(C)** Infiltration scores of different cell types in the GC environment based on the XCELL method.

Supplementary Figure 3 Correlation scores among the three lncRNAs, FRGs, and activated CD4^+^ T cell infiltration scores.

## Glossary

*A2M-AS1*: A2M Antisense RNA 1
*ALOX5*: Arachidonate 5-Lipoxygenase
*ANGPTL4*: Angiopoietin Like 4
*ATF3*: activating transcription factor 3
*ATG4D*: Autophagy Related 4D Cysteine Peptidase
*C2orf27A*: Chromosome 2 Open Reading Frame 27A
CAFs: cancer associated fibroblasts
*CAV1*: Caveolin 1
*CCR5*: C-C Motif Chemokine Receptor 5
*CCR8*: C-C Motif Chemokine Receptor 8
*CDO1*: Cysteine Dioxygenase Type 1
CTL: cytotoxic T lymphocytes
*CXCR3*: C-X-C Motif Chemokine Receptor 3
*CXCR6*: C-X-C Motif Chemokine Receptor 6
DAVID: the database for annotation, visualization and integrated discovery
FPKM: fragments per kilobase per million
FRGs: ferroptosis-related genes
GC: gastric cancer
GO: gene ontology
*GPX4*: glutathione peroxidase 4
*HNF4A*: hepatocyte nuclear factor 4 alpha
*HSBP1*: heat shock factor binding protein 1
*IL12RB1*: Interleukin 12 Receptor Subunit Beta 1
*IL21R*: Interleukin 21 Receptor
*KLRD1*: Killer Cell Lectin Like Receptor D1
lncRNAs: long non-coding RNAs
*LONP1*: Lon Peptidase 1, Mitochondrial
*MYB*: MYB Proto-Oncogene, Transcription Factor
PPI: Protein–Protein Interaction
*PSAT1*: Phosphoserine Aminotransferase 1
STAD: stomach adenocarcinoma
STRING: search tool for the retrieval of interacting genes/proteins
TCGA: the cancer genome atlas
Th: T helper
TME: tumor microenvironment
TP53: Tumor Protein P53
Treg: regulatory T cells
ZNF667-AS1: ZNF667 Antisense RNA 1 (Head To Head)

## References

[B1] Ahrends T. Busselaar J. Severson T. M. Bąbała N. de Vries E. Bovens A. (2019). CD4+ T Cell Help Creates Memory CD8+ T Cells with Innate and Help-independent Recall Capacities. Nat. Commun. 10 (1), 5531. 10.1038/s41467-019-13438-1 31797935 PMC6892909

[B2] Arumugam M. Sugin Lal Jabaris S. (2021). Recent Preclinical Study Offers a Promising Clue: Role of Regulatory T Cells as Biomarkers in Migraine. Immunol. Lett. 240, 9. 10.1016/j.imlet.2021.09.004 34555365

[B3] Behrens G. Li M. Smith C. M. Belz G. T. Mintern J. Carbone F. R. (2004). Helper T Cells, Dendritic Cells and CTL Immunity. Immunol. Cel Biol 82 (1), 84–90. 10.1111/j.1440-1711.2004.01211.x 14984599

[B4] Bejarano L. Jordāo M. J. C. Joyce J. A. (2021). Therapeutic Targeting of the Tumor Microenvironment. Cancer Discov. 11 (4), 933–959. 10.1158/2159-8290.CD-20-1808 33811125

[B5] Bersuker K. Hendricks J. M. Li Z. Magtanong L. Ford B. Tang P. H. (2019). The CoQ Oxidoreductase FSP1 Acts Parallel to GPX4 to Inhibit Ferroptosis. Nature 575 (7784), 688–692. 10.1038/s41586-019-1705-2 31634900 PMC6883167

[B6] Bos R. Sherman L. A. (2010). CD4+ T-Cell Help in the Tumor Milieu Is Required for Recruitment and Cytolytic Function of CD8+ T Lymphocytes. Cancer Res. 70 (21), 8368–8377. 10.1158/0008-5472.CAN-10-1322 20940398 PMC2970736

[B7] Chang C. P. Han P. (2016). Epigenetic and lncRNA Regulation of Cardiac Pathophysiology. Biochim. Biophys. Acta 1863 (7 Pt B), 1767–1771. 10.1016/j.bbamcr.2016.03.005 26969820 PMC7393981

[B8] Chang W.-J. Du Y. Zhao X. Ma L. Y. Cao G. W. (2014). Inflammation-related Factors Predicting Prognosis of Gastric Cancer. Wjg 20 (16), 4586–4596. 10.3748/wjg.v20.i16.4586 24782611 PMC4000495

[B9] Chen X. Huang Y. Shi D. Nie C. Luo Y. Guo L. (2020). LncRNA ZNF667-AS1 Promotes ABLIM1 Expression by Adsorbing microRNA-1290 to Suppress Nasopharyngeal Carcinoma Cell Progression. Ott 13, 4397–4409. 10.2147/OTT.S245554 PMC7248807 32606725

[B10] Corthay A. (2009). How Do Regulatory T Cells Work? Scand. J. Immunol. 70 (4), 326–336. 10.1111/j.1365-3083.2009.02308.x 19751267 PMC2784904

[B11] Cristescu R. Lee J. Nebozhyn M. Kim K.-M. Ting J. C. Wong S. S. (2015). Molecular Analysis of Gastric Cancer Identifies Subtypes Associated with Distinct Clinical Outcomes. Nat. Med. 21 (5), 449–456. 10.1038/nm.3850 25894828

[B12] Dadaglio G. Fayolle C. Oberkampf M. Tang A. Rudilla F. Couillin I. (2020). IL-17 Suppresses the Therapeutic Activity of Cancer Vaccines through the Inhibition of CD8+ T-Cell Responses. Oncoimmunology 9 (1), 1758606. 10.1080/2162402X.2020.1758606 32923117 PMC7458594

[B13] Dai Y. Xu C. Sun X. Chen X. (2017). Nanoparticle Design Strategies for Enhanced Anticancer Therapy by Exploiting the Tumour Microenvironment. Chem. Soc. Rev. 46 (12), 3830–3852. 10.1039/c6cs00592f 28516983 PMC5521825

[B14] Dixon S. J. Lemberg K. M. Lamprecht M. R. Skouta R. Zaitsev E. M. Gleason C. E. (2012). Ferroptosis: an Iron-dependent Form of Nonapoptotic Cell Death. Cell 149 (5), 1060–1072. 10.1016/j.cell.2012.03.042 22632970 PMC3367386

[B15] Doll S. Freitas F. P. Shah R. Aldrovandi M. da Silva M. C. Ingold I. (2019). FSP1 Is a Glutathione-independent Ferroptosis Suppressor. Nature 575 (7784), 693–698. 10.1038/s41586-019-1707-0 31634899

[B16] Eleftheriadis T. Pissas G. Filippidis G. Liakopoulos V. Stefanidis I. (2021). Reoxygenation Induces Reactive Oxygen Species Production and Ferroptosis in Renal Tubular Epithelial Cells by Activating Aryl Hydrocarbon Receptor. Mol. Med. Rep. 23, 23. 10.3892/mmr.2020.11679 33179104 PMC7684866

[B17] Fang K. Caixia H. Xiufen Z. Zijian G. Li L. (2020). Screening of a Novel Upregulated lncRNA, A2M-AS1, that Promotes Invasion and Migration and Signifies Poor Prognosis in Breast Cancer. Biomed. Res. Int. 2020, 9747826. 10.1155/2020/9747826 32352014 PMC7171613

[B18] Feau S. Garcia Z. Arens R. Yagita H. Borst J. Schoenberger S. P. (2012). The CD4+ T-Cell Help Signal Is Transmitted from APC to CD8+ T-Cells via CD27-CD70 Interactions. Nat. Commun. 3, 948. 10.1038/ncomms1948 22781761 PMC3606886

[B19] Gao J. Chen X. Wei P. Wang Y. Li P. Shao K. (2021). Regulation of Pyroptosis in Cardiovascular Pathologies: Role of Noncoding RNAs. Mol. Ther. - Nucleic Acids 25, 220–236. 10.1016/j.omtn.2021.05.016 34458007 PMC8368762

[B20] Gu Y. Chen Y. Jin K. Cao Y. Liu X. Lv K. (2020). Intratumoral CD103+CD4+ T Cell Infiltration Defines Immunoevasive Contexture and Poor Clinical Outcomes in Gastric Cancer Patients. Oncoimmunology 9 (1), 1844402. 10.1080/2162402X.2020.1844402 33312758 PMC7714530

[B21] Hao S. Yu J. He W. Huang Q. Zhao Y. Liang B. (2017). Cysteine Dioxygenase 1 Mediates Erastin-Induced Ferroptosis in Human Gastric Cancer Cells. Neoplasia 19 (12), 1022–1032. 10.1016/j.neo.2017.10.005 29144989 PMC5686465

[B22] Hassannia B. Vandenabeele P. Vanden Berghe T. (2019). Targeting Ferroptosis to Iron Out Cancer. Cancer Cell 35 (6), 830–849. 10.1016/j.ccell.2019.04.002 31105042

[B23] Li W. Li W. Leng Y. Xiong Y. Xia Z. (2020). Ferroptosis Is Involved in Diabetes Myocardial Ischemia/Reperfusion Injury through Endoplasmic Reticulum Stress. DNA Cel Biol. 39 (2), 210–225. 10.1089/dna.2019.5097 31809190

[B24] Li Y. J. Yang Z. Wang Y. Y. Wang Y. (2019). Long Noncoding RNA ZNF667‐AS1 Reduces Tumor Invasion and Metastasis in Cervical Cancer by Counteracting microRNA‐93‐3p‐dependent PEG3 Downregulation. Mol. Oncol. 13 (11), 2375–2392. 10.1002/1878-0261.12565 31420931 PMC6822248

[B25] Liang J.-Y. Wang D.-S. Lin H.-C. Chen X.-X. Yang H. Zheng Y. (2020). A Novel Ferroptosis-Related Gene Signature for Overall Survival Prediction in Patients with Hepatocellular Carcinoma. Int. J. Biol. Sci. 16 (13), 2430–2441. 10.7150/ijbs.45050 32760210 PMC7378635

[B26] Liu Y. Zhang Q. Wu J. Zhang H. Li X. Zheng Z. (2020). Long Non-coding RNA A2M-AS1 Promotes Breast Cancer Progression by Sponging microRNA-146b to Upregulate MUC19. Ijgm 13, 1305–1316. 10.2147/IJGM.S278564 PMC7708314 33273850

[B27] Liu Y. Zhang X. Zhang J. Tan J. Li J. Song Z. (2020). Development and Validation of a Combined Ferroptosis and Immune Prognostic Classifier for Hepatocellular Carcinoma. Front. Cel Dev. Biol. 8, 596679. 10.3389/fcell.2020.596679 PMC7785857 33425905

[B28] Ma R. Shimura T. Yin C. Okugawa Y. Kitajima T. Koike Y. (2021). Antitumor Effects of Andrographis via Ferroptosis-associated G-enes in G-astric C-ancer. Oncol. Lett. 22 (1), 523. 10.3892/ol.2021.12784 34025790 PMC8130053

[B29] Mullarky E. Lucki N. C. Beheshti Zavareh R. Anglin J. L. Gomes A. P. Nicolay B. N. (2016). Identification of a Small Molecule Inhibitor of 3-phosphoglycerate Dehydrogenase to Target Serine Biosynthesis in Cancers. Proc. Natl. Acad. Sci. USA 113 (7), 1778–1783. 10.1073/pnas.1521548113 26831078 PMC4763784

[B30] Nishikawa H. Koyama S. (2021). Mechanisms of Regulatory T Cell Infiltration in Tumors: Implications for Innovative Immune Precision Therapies. J. Immunother. Cancer 9, e002591. 10.1136/jitc-2021-002591 34330764 PMC8327843

[B31] Park S.-H. Cho G. Park S.-G. (2014). NF-κB Activation in T Helper 17 Cell Differentiation. Immune Netw. 14 (1), 14–20. 10.4110/in.2014.14.1.14 24605076 PMC3942503

[B32] Paudel S. Mehtani D. Puri N. (2019). Mast Cells May Differentially Regulate Growth of Lymphoid Neoplasms by Opposite Modulation of Histamine Receptors. Front. Oncol. 9, 1280. 10.3389/fonc.2019.01280 31824856 PMC6881378

[B33] Rowe A. M. Murray S. E. Raué H.-P. Koguchi Y. Slifka M. K. Parker D. C. (2013). A Cell-Intrinsic Requirement for NF-Κb-Inducing Kinase in CD4 and CD8 T Cell Memory. J.I. 191 (7), 3663–3672. 10.4049/jimmunol.1301328 PMC3815446 24006459

[B34] Russick J. Joubert P. E. Gillard-Bocquet M. Torset C. Meylan M. Petitprez F. (2020). Natural Killer Cells in the Human Lung Tumor Microenvironment Display Immune Inhibitory Functions. J. Immunother. Cancer 8, 8. 10.1136/jitc-2020-001054 PMC7570244 33067317

[B35] Schenkel J. M. Zloza A. Li W. Narasipura S. D. Al-Harthi L. (2010). β-Catenin Signaling Mediates CD4 Expression on Mature CD8+T Cells. J.I. 185 (4), 2013–2019. 10.4049/jimmunol.0902572 PMC3963465 20631314

[B36] Shankaraiah R. C. Veronese A. Sabbioni S. Negrini M. (2018). Non-coding RNAs in the Reprogramming of Glucose Metabolism in Cancer. Cancer Lett. 419, 167–174. 10.1016/j.canlet.2018.01.048 29366802

[B37] Song X.-L. Zhang F.-F. Wang W.-J. Li X.-N. Dang Y. Li Y.-X. (2020). LncRNA A2M-AS1 Lessens the Injury of Cardiomyocytes Caused by Hypoxia and Reoxygenation via Regulating IL1R2. Genes Genom 42 (12), 1431–1441. 10.1007/s13258-020-01007-6 33057899

[B38] Stockwell B. R. Friedmann Angeli J. P. Bayir H. Bush A. I. Conrad M. Dixon S. J. (2017). Ferroptosis: A Regulated Cell Death Nexus Linking Metabolism, Redox Biology, and Disease. Cell 171 (2), 273–285. 10.1016/j.cell.2017.09.021 28985560 PMC5685180

[B39] Subhash V. V. Yeo M. S. Tan W. L. Yong W. P. (2015). Strategies and Advancements in Harnessing the Immune System for Gastric Cancer Immunotherapy. J. Immunol. Res. 2015, 308574. 10.1155/2015/308574 26579545 PMC4633567

[B40] Sun X. Liu S. Wang D. Zhang Y. Li W. Guo Y. (2017). Colorectal Cancer Cells Suppress CD4+ T Cells Immunity through Canonical Wnt Signaling. Oncotarget 8 (9), 15168–15181. 10.18632/oncotarget.14834 28147310 PMC5362476

[B41] Sung H. Ferlay J. Siegel R. L. Laversanne M. Soerjomataram I. Jemal A. (2021). Global Cancer Statistics 2020: GLOBOCAN Estimates of Incidence and Mortality Worldwide for 36 Cancers in 185 Countries. CA A. Cancer J. Clin. 71 (3), 209–249. 10.3322/caac.21660 33538338

[B42] Voron T. Marcheteau E. Pernot S. Colussi O. Tartour E. Taieb J. (2014). Control of the Immune Response by Pro-angiogenic Factors. Front. Oncol. 4, 70. 10.3389/fonc.2014.00070 24765614 PMC3980099

[B43] Wang H. Liu C. Zhao Y. Zhang W. Xu K. Li D. (2020). Inhibition of LONP1 Protects against Erastin-Induced Ferroptosis in Pancreatic Ductal Adenocarcinoma PANC1 Cells. Biochem. Biophysical Res. Commun. 522 (4), 1063–1068. 10.1016/j.bbrc.2019.11.187 31822343

[B44] Wang L. Zhang Z. Li M. Wang F. Jia Y. Zhang F. (2019). P53-dependent Induction of Ferroptosis Is Required for Artemether to Alleviate Carbon Tetrachloride-Induced Liver Fibrosis and Hepatic Stellate Cell Activation. IUBMB Life 71 (1), 45–56. 10.1002/iub.1895 30321484

[B45] Wang N. Feng Y. Xie J. Han H. Dong Q. Wang W. (2020). Long Non-coding RNA ZNF667-AS1 Knockdown Curbs Liver Metastasis in Acute Myeloid Leukemia by Regulating the microRNA-206/AKAP13 Axis. Cmar 12, 13285–13300. 10.2147/CMAR.S269258 PMC7767707 33380835

[B46] Wang S.-J. Li D. Ou Y. Jiang L. Chen Y. Zhao Y. (2016). Acetylation Is Crucial for P53-Mediated Ferroptosis and Tumor Suppression. Cel Rep. 17 (2), 366–373. 10.1016/j.celrep.2016.09.022 PMC5227654 27705786

[B47] Wang W. Green M. Choi J. E. Gijón M. Kennedy P. D. Johnson J. K. (2019). CD8+ T Cells Regulate Tumour Ferroptosis during Cancer Immunotherapy. Nature 569 (7755), 270–274. 10.1038/s41586-019-1170-y 31043744 PMC6533917

[B48] Wei M. Shen D. Mulmi Shrestha S. Liu J. Zhang J. Yin Y. (2018). The Progress of T Cell Immunity Related to Prognosis in Gastric Cancer. Biomed. Res. Int. 2018, 3201940. 10.1155/2018/3201940 29682534 PMC5848132

[B49] Yao M. Ventura P. B. Jiang Y. Rodriguez F. J. Wang L. Perry J. S. A. (2020). Astrocytic Trans-differentiation Completes a Multicellular Paracrine Feedback Loop Required for Medulloblastoma Tumor Growth. Cell 180 (3), 502–520. 10.1016/j.cell.2019.12.024 31983537 PMC7259679

[B50] Yao Y. Chen Z. Zhang H. Chen C. Zeng M. Yunis J. (2021). Selenium-GPX4 axis Protects Follicular Helper T Cells from Ferroptosis. Nat. Immunol. 22 (9), 1127–1139. 10.1038/s41590-021-00996-0 34413521

[B51] Yuan L. Xu B. Yuan P. Zhou J. Qin P. Han L. (2017). Tumor-infiltrating CD4+ T Cells in Patients with Gastric Cancer. Cancer Cel Int 17, 114. 10.1186/s12935-017-0489-4 PMC5712164 29213216

[B52] Yuan W. Tao R. Huang D. Yan W. Shen G. Ning Q. (2021). Transcriptomic Characterization Reveals Prognostic Molecular Signatures of Sorafenib Resistance in Hepatocellular Carcinoma. Aging 13 (3), 3969–3993. 10.18632/aging.202365 33495404 PMC7906139

[B53] Zhang H. Deng T. Liu R. Ning T. Yang H. Liu D. (2020). CAF Secreted miR-522 Suppresses Ferroptosis and Promotes Acquired Chemo-Resistance in Gastric Cancer. Mol. Cancer 19 (1), 43. 10.1186/s12943-020-01168-8 32106859 PMC7045485

[B54] Zhang X. Du L. Qiao Y. Zhang X. Zheng W. Wu Q. (2019). Ferroptosis Is Governed by Differential Regulation of Transcription in Liver Cancer. Redox Biol. 24, 101211. 10.1016/j.redox.2019.101211 31108460 PMC6526247

[B55] Zhuang L. Ding W. Ding W. Zhang Q. Xu X. Xi D. (2021). lncRNA ZNF667‐AS1 (NR_036521.1) Inhibits the Progression of Colorectal Cancer via Regulating ANK2/JAK2 Expression. J. Cel Physiol 236 (3), 2178–2193. 10.1002/jcp.30004 32853419

