# Supplementary figures and images for: LncRNAs Target Ferroptosis-Related Genes and Impair Activation of CD4+ T Cell in Gastric Cancer
Source: Front Cell Dev Biol. 2021 Dec 13;9:797339. doi: 10.3389/fcell.2021.797339 (PMC8710671; doi:10.3389/fcell.2021.797339)

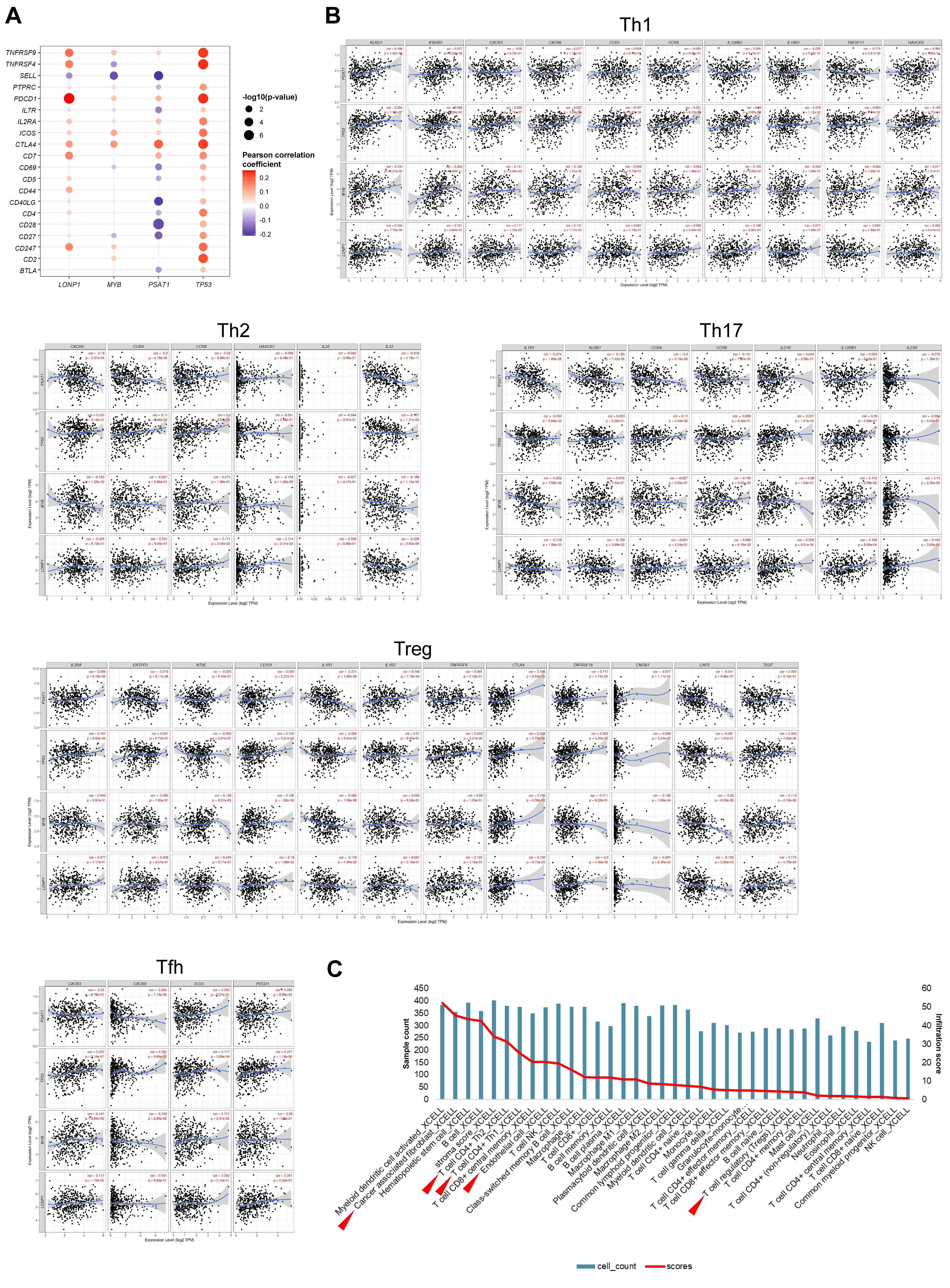

Supplement: Supplementary file 4 [file Image3.TIF]

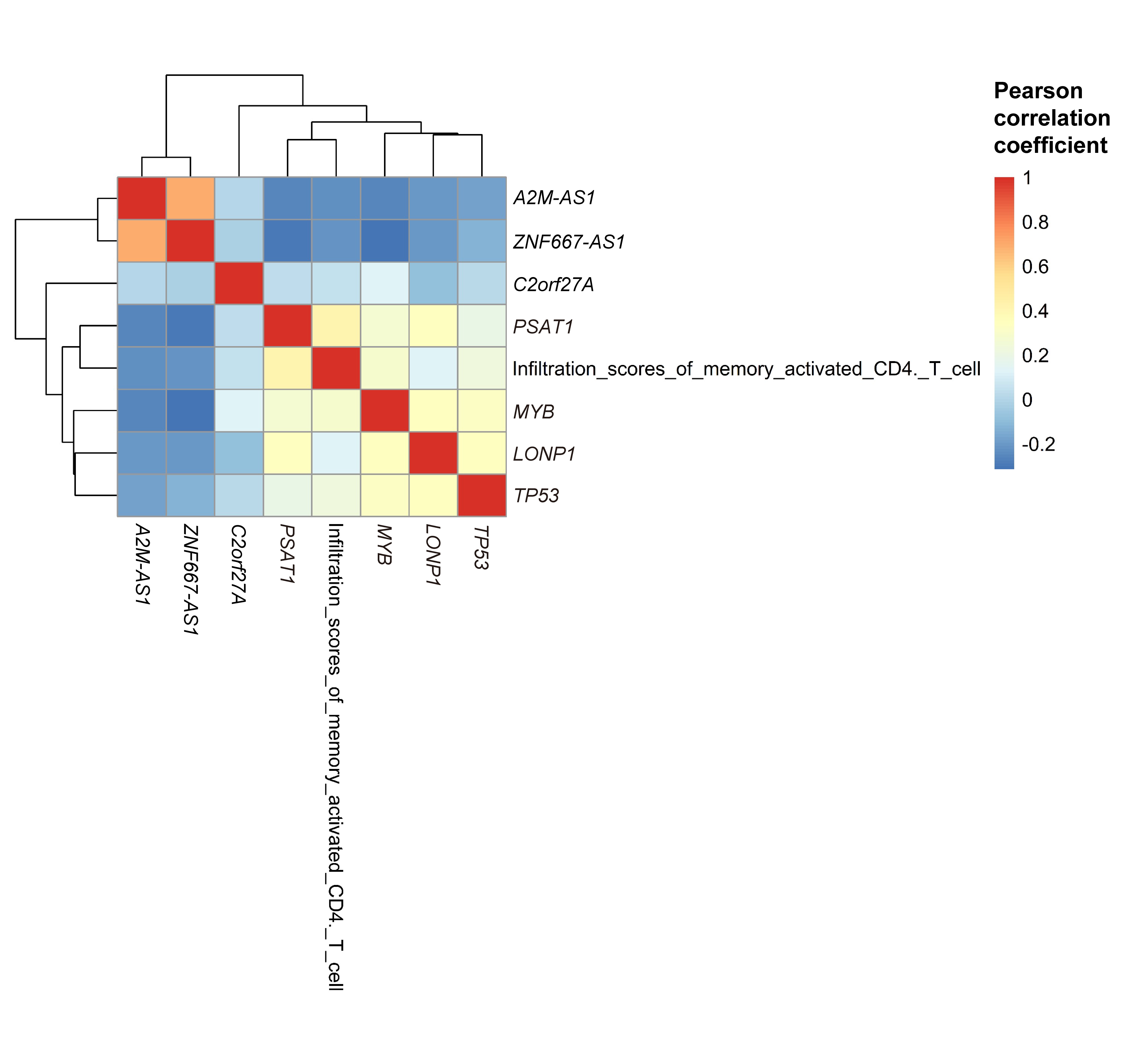

Supplement: Supplementary file 5 [file Image2.TIF]

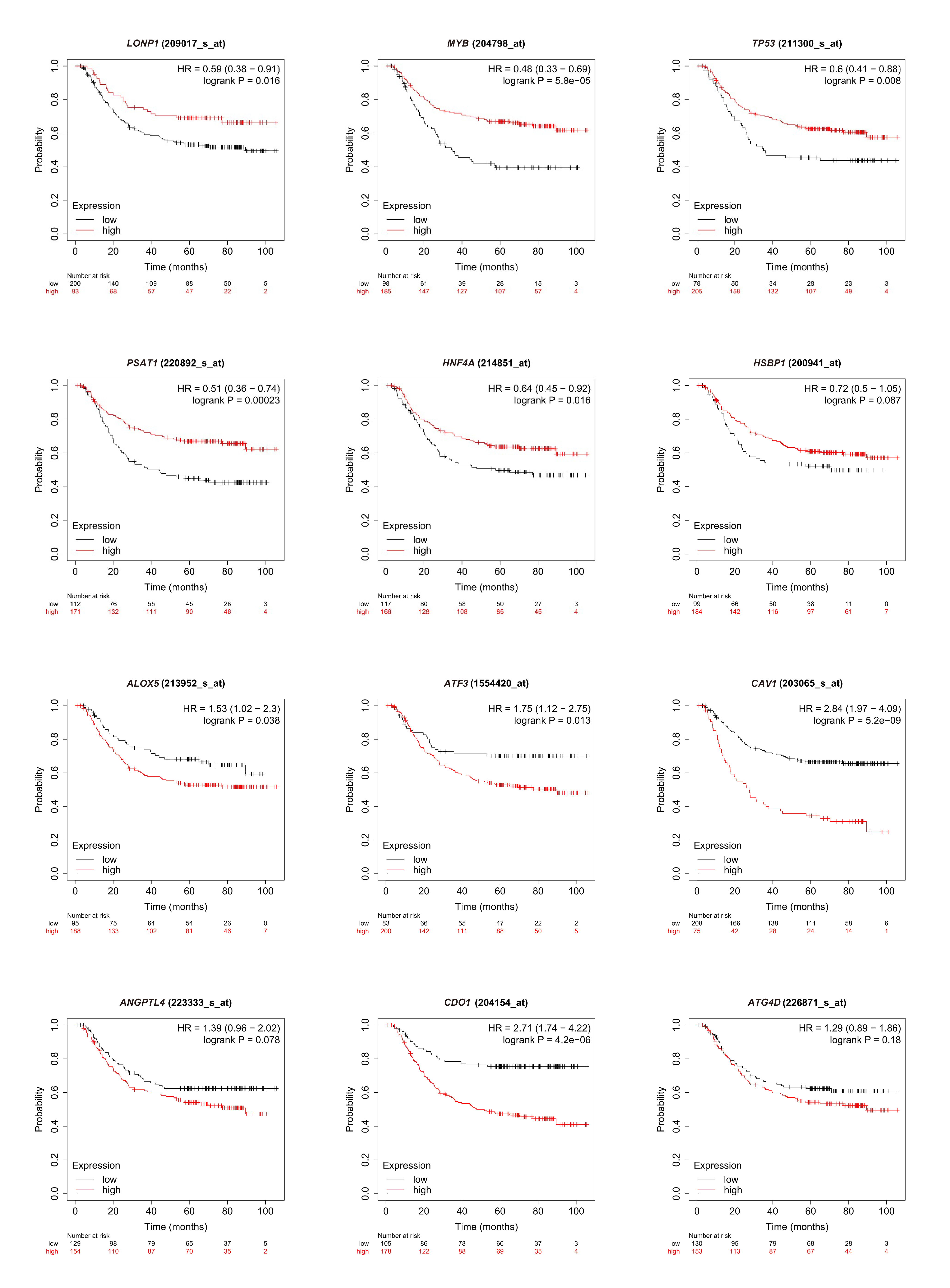

Supplement: Supplementary file 6 [file Image1.TIF]
